# Uncovering a Role for the Dorsal Hippocampal Commissure in Recognition Memory

**DOI:** 10.1093/cercor/bhz143

**Published:** 2019-07-29

**Authors:** M Postans, G D Parker, H Lundell, M Ptito, K Hamandi, W P Gray, J P Aggleton, T B Dyrby, D K Jones, M Winter

**Affiliations:** 1 Cardiff University Brain Research Imaging Centre, CF24 4HQ; 2 School of Psychology, CF10 3AS; 3 Experimental MRI Centre, School of Biosciences, Cardiff University, Cardiff CF10 3AX, UK; 4 Danish Research Centre for Magnetic Resonance, Centre for Functional and Diagnostic Imaging and Research, Copenhagen University Hospital Hvidovre, Hvidovre, DK-2650, Denmark; 5 School of Optometry, University of Montreal, H3T 1J4 Montreal, Canada; 6 Department of Neurology and Neurosurgery, Montreal Neurological Institute, H3A 2B4 Montreal, Canada; 7 The Alan Richens Welsh Epilepsy Centre, Department of Neurology, University Hospital of Wales, Cardiff CF14 4XW, UK; 8 Institute of Psychological Medicine and Clinical Neurosciences; 9 Brain Repair And Intracranial Neurotherapeutics Unit, School of Medicine, Cardiff University, Cardiff CF24 4HQ, UK; 10 Department of Neurosurgery, Neurosciences Division, University Hospital Wales, Cardiff, CF14 4XW, UK; 11 Department of Applied Mathematics and Computer Science, Technical University of Denmark, Kongens Lyngby, Denmark, DK-2800; 12 Mary MacKillop Institute for Health Research, Australian Catholic University, Melbourne 3000, Australia; 13 Department of Clinical Neuropsychology, University Hospital of Wales, Cardiff, CF14 4XW, UK

**Keywords:** familiarity, hippocampal commissure, recognition memory, recollection, tractography

## Abstract

The dorsal hippocampal commissure (DHC) is a white matter tract that provides interhemispheric connections between temporal lobe brain regions. Despite the importance of these regions for learning and memory, there is scant evidence of a role for the DHC in successful memory performance. We used diffusion-weighted magnetic resonance imaging (DW-MRI) and white matter tractography to reconstruct the DHC in both humans (in vivo) and nonhuman primates (ex vivo). Across species, our findings demonstrate a close consistency between the known anatomy and tract reconstructions of the DHC. Anterograde tract-tracer techniques also highlighted the parahippocampal origins of DHC fibers in nonhuman primates. Finally, we derived diffusion tensor MRI metrics from the DHC in a large sample of human subjects to investigate whether interindividual variation in DHC microstructure is predictive of memory performance. The mean diffusivity of the DHC correlated with performance in a standardized recognition memory task, an effect that was not reproduced in a comparison commissure tract—the anterior commissure. These findings highlight a potential role for the DHC in recognition memory, and our tract reconstruction approach has the potential to generate further novel insights into the role of this previously understudied white matter tract in both health and disease.

## Introduction

The 2 hemispheres of the brain are connected by commissural fiber systems that include the corpus callosum, anterior commissure (AC), posterior commissure, ventral hippocampal commissure (VHC), and dorsal hippocampal commissure (DHC) (Demeter et al. 1985). The DHC (alternatively the “dorsal psaltarium”) provides interhemispheric connections between functionally-related structures in the medial temporal lobes (MTLs), including the presubiculum and entorhinal and parahippocampal cortices (Demeter et al. 1985, 1990; Gloor et al. 1993). Given that these regions play a key role in successful learning and memory (Zola-Morgan et al. 1989; Squire and Zola-Morgan 1991; Aggleton and Brown 1999; Aggleton 2012), their ability to communicate effectively with contralateral homologous regions via the DHC may also be important for performance in these cognitive domains.

There have, however, been few studies of the function of the DHC, potentially due to misunderstanding around the cross-species anatomy of the DHC, as distinct from other local fiber populations such as the VHC, fornix, and corpus callosum (Demeter et al. 1985; Raslau et al. 2015; Tubbs et al. 2015). Confusion also arises as some authors distinguish the commissural fibers in the inferior forceps of the corpus callosum from those of the DHC, whereas others do not make this distinction (Demeter et al. 1985). Additional confusion has arisen from the occasional mislabeling of the DHC as the VHC (e.g., Paxinos et al. 2009). In rodents, the VHC supports dense interhemispheric connections between the hippocampi, which originate throughout the long axis of the hippocampus; in nonhuman primates, VHC connections are reduced so that only the uncal and genual subdivisions of the hippocampal formation are connected to those in the contralateral hemisphere (Demeter et al. 1985; Gloor et al. 1993). By contrast, the DHC remains a substantial tract in nonhuman primates, but it carries commissural projections to and from the parahippocampal region rather than the hippocampus proper. From injection sites in the presubiculum and entorhinal and parahippocampal cortices, tract-tracer studies in nonhuman primates have traced labeled DHC fibers into the alveus (Demeter et al. 1985, 1990). Without entering the fimbria–fornix itself, these fibers continue to travel through the alveus towards the hippocampus tail, at which point they become attached to the inferior surface of the posterior columns (crus) of the fornix. From here, these fibers arch dorso-anteriorly and then turn medially to cross the midline along the inferior aspect of the corpus callosum, before taking a mirror-image route back to the contralateral parahippocampal region (Demeter et al. 1985, 1990). Anatomical studies have found no convincing evidence of a VHC in humans, but the location of the human DHC corresponds precisely to that reported for nonhuman primates (Gloor et al. 1993). Despite their distinct anatomy, the VHC and DHC are sometimes collectively termed “the hippocampal commissure” (Demeter et al. 1985), and the DHC is sometimes described as part of the fornix (e.g., “fornix commissure”) (Mark et al. 1993). It is, however, difficult to infer the function of the DHC from potentially informative clinical case reports and animal studies if it is not appropriately differentiated from these other structures.

In one relevant study highlighting a potential role for the DHC in successful discrimination learning, fornix transection did not impair the ability of monkeys to learn concurrent visual object discriminations, but the fornix damage in one subject extended to the DHC, and that subject made significantly more errors and required more training sessions to learn the task compared with the slowest control (Moss et al. 1981). This subject was also impaired in a visual object recognition memory task (Mahut et al. 1981). Similarly, clinical case reports describe individuals with anterograde amnesia following combined DHC and fornix damage (Heilman and Sypert 1977; D’Esposito et al. 1995), although it is difficult to evaluate the effect of DHC damage in these cases because fornix damage alone is sufficient to produce anterograde amnesia (Aggleton 2008). A deficit in both verbal and visual recall has also been reported in patients who underwent callosotomy surgery for intractable epilepsy but only when the section included the posterior corpus callosum (Clark and Geffen 1989; Phelps et al. 1991). This is pertinent because the rostral splenium and posterior DHC fibers are intermingled, so split-brain surgery involving the posterior corpus callosum always involves DHC transection.

The inferences we can derive from these small, methodologically heterogenous studies are, however, limited. Patients with verifiable DHC damage are extremely rare, and there are no reported cases of DHC damage sparing other relevant structures. An alternative approach is to examine whether interindividual variation in the microstructure of the DHC is related to differences in memory performance. Begré et al. used diffusion tensor magnetic resonance imaging (DT-MRI) to search, voxel-wise, for a correlation between a measure of white matter microstructure (intervoxel coherence) and performance in the Rey Visual Design Learning Test (Begré et al. 2009). In their small sample (*N* = 14), the authors reported that clusters of voxels demonstrating such a relationship overlapped with the DHC. The reported coordinates, however, correspond to the inferior-caudal surface of the splenium, whereas histological studies localize the DHC ventral to the corpus callosum body, with posterior DHC fibers becoming intermingled with those of the rostral splenium (Demeter et al. 1990). The clusters reported by Begré et al. may therefore lack specificity to the DHC. Wei et al. (2017) recently demonstrated that white matter tractography and diffusion-weighted MRI (DW-MRI) can be used to isolate and reconstruct the trajectory of the human DHC, in vivo, but no individual subject-level reconstructions were shown (a group-level reconstruction was provided), and the study did not investigate the relationship between DHC microstructure and cognitive performance. A study in a larger sample is therefore required to isolate the human DHC systematically and investigate the functional role of this tract in memory. Evidence that the DHC can be reconstructed accurately in nonhuman primates, where the tract morphology has been well characterized, would reinforce confidence in the accuracy of human DHC reconstructions.

In the present study, we report a semiautomated tractography approach that can be used to reconstruct the DHC across humans (in vivo) and nonhuman primates (ex vivo). We also present tract-tracer findings highlighting that primate DHC fibers form a distinct tract and originate in the parahippocampal region rather than the hippocampal formation. Finally, we derived DT-MRI metrics from the DHC in a large sample of 100 human subjects to investigate whether interindividual variation in the microstructure of this tract correlates with memory performance. We also assessed whether the bilateral volumes of several relevant gray matter MTL regions relate to memory performance.

## Materials and Methods

### Data

#### Ex Vivo Nonhuman Primate Magnetic Resonance Data

Diffusion- and *T*_1_-weighted magnetic resonance (MR) data obtained previously from the perfusion-fixed brains of 4 healthy adult female vervet monkeys (*Chlorocebus sabeus*; specimens e3429, e3487, e3494, and e4271) were available for analysis (age range = 32–48 months; mean = 36.25, standard deviation [SD] = 7.85). The animals were obtained from the Behavioral Science Foundation, St. Kitts, and were socially housed in enriched environments. The experimental procedures were reviewed and approved by the institutional review board of the Behavioral Science Foundation, acting under the auspices of the Canadian Council on Animal Care. The postmortem brains were prepared for data collection on a preclinical 4.7-T Agilent scanner system at the Danish Research Centre for Magnetic Resonance using an ex vivo imaging protocol reported previously (Dyrby et al. 2011, 2014). This included a DW-MRI prescan of at least 15 h in duration to avoid introducing short-term instabilities into the final DW-MRI datasets (e.g., due to motion caused by physical handling of the tissue; Dyrby et al. 2011, 2013). The brain specimens were also stabilized to room temperature prior to scanning, and a conditioned flow of air around the specimen was maintained throughout scanning to reduce temperature drifts of the diffusion signal (Dyrby et al. 2011, 2013).

Diffusion-weighted images were collected using a diffusion-weighted pulsed gradient spin echo sequence with single-line readout. The scan parameters were as follows: repetition time, TR = 7200 ms (but TR = 8400 ms for subject e4271); echo time, TE = 35.9 ms; gradient separation, delta = 17.0 ms; gradient duration, delta = 10.5 ms; gradient strength, *g* = 300 mT/m; number of repetitions, NEX = 2 (averaged offline); matrix size = 128 × 256 with 100 axial slices offering whole-brain coverage with isotropic 0.5-mm voxels. Gradients were applied along 68 uniformly distributed directions with a *b* value of 9686 s/mm^2^ using scheme files available from the Camino tool kit (Cook et al. 2006). Thirteen non–diffusion-weighted images with *b* = 0 s/mm^2^ were also acquired. *T*_1_-weighted images were acquired using a 3D magnetization prepared rapid gradient echo (MPRAGE) sequence with 0.27-mm isotropic voxels and the following parameters: TR = 4 ms, TE = 2 ms, time to inversion (TI) = 800 ms, flip angle (FA) = 9°, matrix = 256 × 256 × 256, and axial image plane.

#### Ex Vivo Nonhuman Primate Anterograde Tract-Tracer Data

To highlight the distinct origins of fibers comprising the DHC and the nearby fornix, we examined ex vivo brain specimens obtained from 3 male cynomolgus monkeys (*Macaca fascicularis*: ACy14, ACyF23, and ACy28) aged 1–2 years that had received anterograde tract-tracer injections in different MTL regions for a previous study of the origin and topography of the fibers comprising the fornix (Saunders and Aggleton 2007).

For further details of the stereotactic surgeries, the reader is referred to the original description (Saunders and Aggleton 2007), but briefly, a cocktail of tritiated amino acids was injected into distinct target regions within the MTL. This cocktail was composed of an equal-parts mixture of tritiated proline and leucine (a final concentration of 50 μCi/μL; New England Nuclear) and was injected using a 1-μL Hamilton syringe. Case ACy28 received 3 injections of amino acids (0.41 μL in total) that together largely filled all fields of the posterior hippocampus, including the subiculum, and just reached the adjacent part of the presubiculum. Case ACy14 received a single injection (0.14 μL) in the hippocampal formation, centered in the subiculum in the rostral hippocampus, leveled with the caudal half of the uncus. Finally, case ACyF23 received a single injection (0.14 μL) that incorporated the caudal perirhinal cortex and the rostral parahippocampal cortex (area 35). Following 6–7 days of postoperative survival, the 3 monkeys were deeply anesthetized, and their brains were removed and cryoprotected. The tissue was cut into 33-μm coronal sections, coated with emulsion, and subsequently exposed at 4 °C for 6–20 weeks before being developed and counterstained for thionine (Aggleton et al. 1986). Case ACyF23 had undergone a bilateral fornix transection procedure 9 months prior to the injection of the tritiated amino acids; the case is nevertheless informative as the transection was rostral to the DHC and because the subsequent amino acid injections resulted in labeling up to the point of the fornix transection, that is, the fibers still contained an anterogradely transported label.

#### In Vivo Human MR and Cognitive Data

Cognitive, diffusion- and *T*_1_-weighted MR data were obtained for 100 subjects from the Q3 release of the Human Connectome Project (HCP; Glasser et al. 2013; Sotiropoulos et al. 2013; Van Essen et al. 2013). One hundred subjects with consecutive HCP subject identifier codes were selected for this subsample, ensuring that all required data were available across all subjects and there was an even representation of males and females (50 males, aged 22–35 years). The participants in that previous study were recruited from Washington University and the surrounding area and gave informed consent in line with policies approved by the Washington University Institutional Review Board. We co-opted these data for the present analyses to exploit the high-quality diffusion-weighted images that are acquired through the HCP owing to the superior gradient strengths afforded by their customized gradient set. This subsample of the available HCP data will henceforth be referred to as the “HCP dataset.” For each subject, whole-brain diffusion- and *T*_1_-weighted images had been acquired on a customized 3-T Connectom Skyra scanner (Siemens, Erlangen) with a 32-channel head coil and a customized SC72C gradient set. Each preprocessed dataset comprised 90 diffusion directions for each of 3 shells with *b* values of 1000, 2000, and 3000 s/mm^2^; these images were acquired with TR = 5500 ms, TE = 89 ms, and 1.25 × 1.25 × 1.25 mm^3^ isotropic voxels. Eighteen images with *b* = 0 s/mm^2^ were also acquired. Corresponding *T*_1_-weighted images were acquired by taking 2 averages using the 3D MPRAGE sequence (Mugler and Brookeman 1990), with 0.7 × 0.7 × 0.7 mm^3^ isotropic voxels and the following parameters: TR = 2400 ms, TE = 2.14 ms, TI = 1000 ms, FA = 8°, field of view (FOV) = 224 mm, matrix = 320 × 320 × 256 sagittal slices in a single slab. Note that the preprocessed HCP diffusion datasets are aligned to the *T*_1_-weighted images using FLIRT (Jenkinson and Smith 2001; Jenkinson et al. 2002) as standard so that both the diffusion- and *T*_1_-weighted data that were available to us were prealigned in a 1.25-mm native structural space. Further acquisition parameters and details of the minimal MR preprocessing pipeline have been reported previously (Glasser et al. 2013; Sotiropoulos et al. 2013). The anatomical scans were inspected to confirm the images did not contain obvious anatomical abnormalities.

Available cognitive data for the HCP subjects included performance in the Computerized Penn Word Memory (CPWM) task (Moore et al. 2015), the Picture Sequence Memory Test (PSMT; Dikmen et al. 2014), and the List Sorting Working Memory Test (LSWMT; Tulsky et al. 2014). The CPWM is a verbal recognition memory task in which a participant is required to discriminate 20 pre-exposed target word stimuli from 20 intermixed novel distractor stimuli; performance is quantified here as subjects' total number of correct responses. The PSMT is an episodic memory task in which subjects are required to learn and recall a sequence of picture stimuli over a number of trials and performance is scored as the cumulative number of adjacent pairs of pictures that are correctly recalled over 3 learning trials. In the LSWMT, subjects are presented with a series of picture stimuli on a computer screen (e.g., an elephant and a mouse) and are required to remember the stimuli comprising the sequence, mentally reorder them from smallest to largest, and finally recite the revised sequence of stimuli; performance is scored as the number of correct responses across the stimulus lists that comprise this working memory task. For both the PSMT and LSWMT, HCP subjects' raw scores have been standardized against the NIH Toolbox normative sample (Weintraub et al. 2013). These standardized scores can also be age-adjusted, but given that we had non–age-adjusted raw scores for the CPWM, we used subjects' unadjusted PSMT and LSWMT scores for subsequent analyses.

Finally, pre-existing regional volume measures were available for a number of relevant cortical and subcortical regions in the HCP dataset, because the *T*_1_- and *T*_2_-weighted images that are acquired for the HCP are segmented using FreeSurfer software as part of the standard preprocessing pipeline (Glasser et al. 2013). We used these data to investigate whether differences in CPWM, PSMT, and/or LSWMT performance are also related to the volume of several key gray matter regions within the MTL. Our specific regions-of-interest (ROIs) were the hippocampi, amygdalae, entorhinal cortex, and parahippocampal cortex (areas TH and TF), as well as the temporal pole, and estimates of total intracranial volume (ICV). The hippocampus was of interest because the DHC is sometimes assumed to support dense interhippocampal connections, despite an absence of confirmatory evidence (Demeter et al. 1990). By contrast, the entorhinal and parahippocampal cortices are known to project to contralateral structures via the DHC (Demeter et al. 1985, 1990), and they also provide a functionally important input/output pathway for the hippocampus itself (Aggleton 2012). The temporal pole and amygdala were ROIs that are known to project to or receive from contralateral structures via the AC, which was used as a comparison tract for our tractography analyses, as described below (Klingler and Gloor 1960; Turner et al. 1979; Demeter et al. 1985).

### Data Processing

#### Ex Vivo Nonhuman Primate MR Data

The *T*_1_-weighted images for each nonhuman primate specimen were masked to contain only brain tissue using FSL utilities (Smith et al. 2004). Gray/white matter contrast is reversed in our *T*_1_-weighted images of ex vivo tissue (Dyrby et al. 2018); we therefore inverted the *T*_1_-weighted images for subsequent processing and display purposes. The *T*_1_-weighted brain images were then submitted to the standard Nonhuman Primate EMSegmenter pipeline in 3DSlicer version 4.9.0. The pipeline registers the *T*_1_-weighted image to a probabilistic vervet monkey MRI atlas using BRAINSFit (Johnson et al. 2007; Fedorov et al. 2011) and segments the image into unilateral ROIs, including the hippocampus, using the EMSegmenter algorithm (Pohl et al. 2007). The subject-specific aligned and unbiased hippocampus segmentations were thresholded at 40%, binarized, and brought into native diffusion space using FLIRT, ready for use as ROIs for tractography.

**Figure 1:**
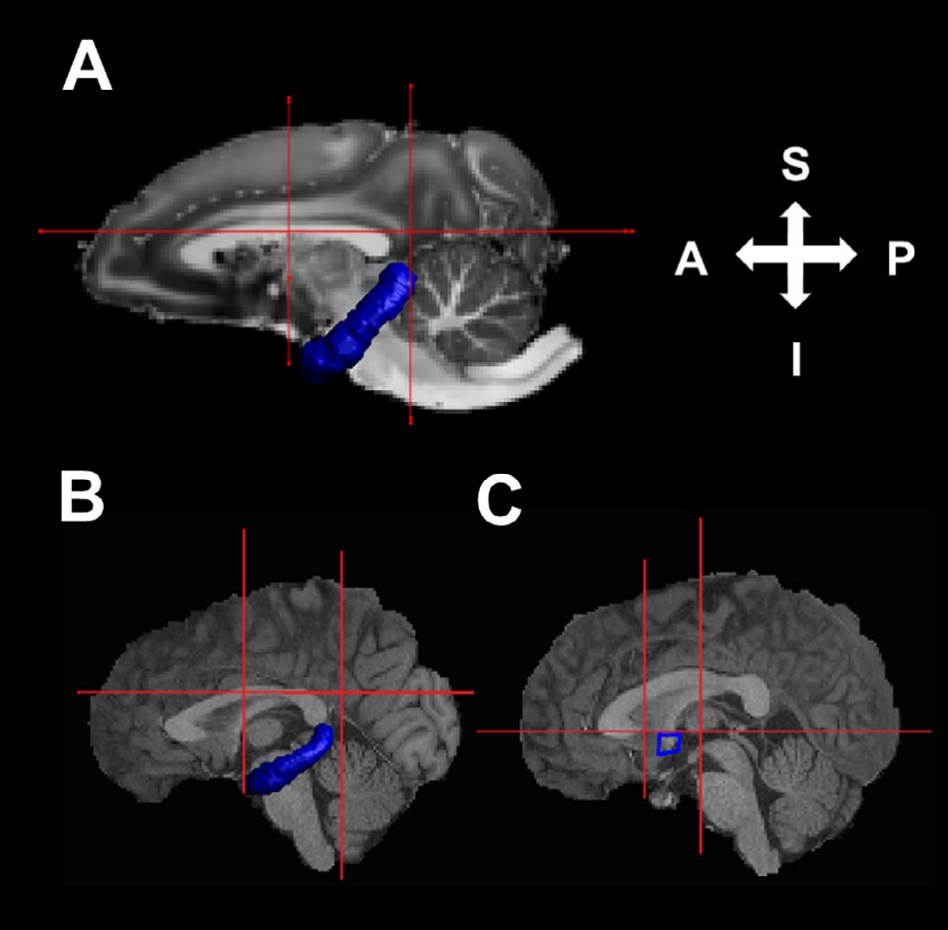
ROIs used for DHC and AC tractography. The hippocampal (blue) and manually drawn (red lines) ROIs used for DHC tractography shown on a midsagittal section of a *T*_1_-weighted image for a representative ex vivo nonhuman primate specimen in a 0.5-mm^3^ native diffusion space (*A*) and an HCP subject in a 1.25-mm^3^ native diffusion space (*B*). Also shown are the manually-drawn ROIs used for AC tractography (red lines and blue square) in a representative HCP subject (*C*).

Visual inspection of the DW-MRI datasets revealed that no additional preprocessing was required to adjust for motion or eddy currents prior to streamline reconstruction (Dyrby et al. 2014). A multiple-ROI tractography approach (see Fig. 1*A*), based on seeding streamlines from subjects' hippocampal ROIs, was used to reconstruct the DHC. The nonhuman primate DHC connects parahippocampal as opposed to hippocampal areas, but these parahippocampal projections aggregate and travel directly through the alveus towards the tail of the hippocampus without entering the fimbria–fornix (Demeter et al. 1985, 1990). The precise cortical origins of the human DHC have not been directly confirmed, but its fibers are likewise known to “merge with the alveus covering the hippocampus” (Gloor et al. 1993). Owing to its contact with the alveus, DHC tractography can therefore be successfully seeded from the hippocampus across both humans and nonhuman primates. Indeed, the DHC is the only commissural fiber bundle in direct contact with the hippocampus, whereas the parahippocampal gyrus is connected to multiple spatially dispersed brain regions (Powell et al. 2004; Yogarajah et al. 2008) and therefore gives rise to several prominent and crossing noncommissural (e.g., the parahippocampal cingulum) and commissural (AC and splenium) fiber populations. This translational approach builds on a previous study in which hippocampal ROIs were used to reconstruct the human DHC (Wei et al. 2017).

Tractography was performed from all voxels in the left hippocampus ROI in subjects' native diffusion space in ExploreDTI v4.8.3 (Leemans et al. 2009) using a deterministic tractography algorithm based on constrained spherical deconvolution (CSD) (Tournier et al. 2008; Jeurissen et al. 2011). The contralateral hippocampal ROI was used as an “AND” gate to capture any propagated streamlines that terminated in the contralateral hippocampal/parahippocampal region. Three additional “NOT” ROIs were manually drawn to exclude streamlines corresponding to other pathways. These included 1) an ROI covering the entire section, drawn on the most inferior axial slice where the body of the corpus callosum was visible; 2) a coronal ROI covering the entire section placed at a slice where the parahippocampal cingulum begins to descend behind the splenium; and 3) a coronal ROI covering the entire section except the temporal lobes, placed at the slice where the anterior fornix columns descend towards the mammillary bodies. Additional exclusionary ROIs were used to remove extant spurious streamlines as required. A step size of 0.1 mm and an angle threshold of 60° were applied to prevent the reconstruction of anatomically implausible streamlines. Tracking was performed with a supersampling factor of 4 × 4 × 4; that is, streamlines were initiated from 64 grid points, uniformly distributed within each voxel.

To illustrate the location of the DHC with respect to the adjacent fornix, we used a previously published multiple-ROI protocol to reconstruct the fornix in representative nonhuman primate (NHP) and HCP subjects (see Metzler-Baddeley et al. 2011). The DHC also has a similar morphology to the tapetum of the corpus callosum; for illustrative purposes, we therefore used a multiple-ROI approach to reconstruct the tapetum in representative NHP and HCP subjects. These ROIs included 2 AND ROIs that were drawn around the splenium on sagittal sections located 5 slices from the midline on each side of the brain (10 slices for the HCP data). Two exclusionary ROIs covering the entire section inferior to the genu and splenium were drawn on the same sagittal sections to preclude the erroneous reconstruction of streamlines that crossed back through other commissural pathways (e.g., the posterior commissure). An exclusionary ROI was placed on an axial section immediately superior to the cingulum; another axial exclusionary ROI was drawn between the lateral ventricles on a section immediately inferior to the splenium. Two final exclusionary ROIs were placed on coronal sections that were 1) immediately anterior to the rostral border of the anterior fornix columns (covered the entire section except the temporal lobes) and 2) immediately posterior to the caudal border of the cingulum as it arches ventrally around the splenium (covered the entire section).

#### Ex Vivo Nonhuman Primate Anterograde Tract-Tracer Data

A Leica DM500B microscope with a Leica DFC310FX digital camera and Leica Application Suite v4.7 image acquisition software was used to obtain both bright- and dark-field images from our ex vivo cynomolgus monkey specimens.

#### In Vivo Human MR and Cognitive Data

Whole-brain voxel-wise maps of 2 DT-MRI measures of white matter microstructure—fractional anisotropy and mean diffusivity (FA and MD, respectively) (Basser and Pierpaoli 2011)—were derived from the *b* = 1000 s/mm^2^ images. Unilateral hippocampal ROIs were segmented from subjects' *T*_1_-weighted images using FIRST (Patenaude et al. 2011). Streamlines were then seeded from the left hippocampus using the same combination of ROIs described above (Fig. 1*B*), and a multishell, multitissue CSD algorithm was applied to the subjects' complete diffusion dataset (Jeurissen et al. 2014). This process was then repeated with tractography seeded from the right hippocampus. Tracking parameters were the same as above except a step size of 0.5 mm was applied. Two additional ROIs were then drawn around the DHC reconstructions on sagittal sections located 5 slices from the midline of the brain to extract a transverse segment of the DHC, where the streamlines are well differentiated from those of other local white matter pathways. This “cutting” approach has been employed in previous studies investigating the relationship between interindividual variation in the structural properties of commissural connections and cognitive task performance (Davis and Cabeza 2015) and was done separately for the reconstructions obtained by seeding tractography from the left and right hemispheres. The transverse DHC segments were intersected with the whole-brain voxel-wise FA and MD maps. For both FA and MD, the mean measures obtained from the 2 segments were then combined into a vertex-weighted mean measure as follows:

$${\displaystyle \begin{array}{l}\ \ \mathrm{Vertex}-\mathrm{Weighted}\ \mathrm{Mean}\ \mathrm{FA}=\frac{\ \left({N}_{\mathrm{L}\to \mathrm{R}}\times \overline{{\mathrm{FA}}_{\mathrm{L}\to \mathrm{R}}}\right)+\left({N}_{\mathrm{R}\to \mathrm{L}}\times \overline{{\mathrm{FA}}_{\mathrm{R}\to \mathrm{L}}}\right)}{\left({N}_{\mathrm{L}\to \mathrm{R}}+{N}_{\mathrm{R}\to \mathrm{L}}\right)}\\[15pt] {}\mathrm{Vertex}-\mathrm{Weighted}\ \mathrm{Mean}\ \mathrm{MD}=\frac{\ \left({N}_{\mathrm{L}\to \mathrm{R}}\times \overline{{\mathrm{MD}}_{\mathrm{L}\to \mathrm{R}}}\right)+\left({N}_{\mathrm{R}\to \mathrm{L}}\times \overline{{\mathrm{MD}}_{\mathrm{R}\to \mathrm{L}}}\right)}{\left({N}_{\mathrm{L}\to \mathrm{R}}+{N}_{\mathrm{R}\to \mathrm{L}}\right)}\end{array}}$$

where *N*_L→R_ and *N*_R→L_ refer to the number of vertices comprising the tract segment obtained by seeding tractography from the left and right hemispheres, respectively. $\overline{{\mathrm{FA}}_{\mathrm{L}\to \mathrm{R}}}$ and $\overline{{\mathrm{FA}}_{\mathrm{R}\to \mathrm{L}}}$ refer to the mean FA measure obtained from the left- and right-seeded segments, respectively; likewise, $\overline{{\mathrm{MD}}_{\mathrm{L}\to \mathrm{R}}}$ and $\overline{{\mathrm{MD}}_{\mathrm{R}\to \mathrm{L}}}$ refer to the mean MD measure obtained from the left- and right-seeded segments, respectively. These vertex-weighted measures of mean FA and MD take into account any potential differences in the number of streamlines that comprise the left- versus right-seeded segments and were later correlated with memory measures. For the sake of brevity, in the remainder of the text, we refer simply to mean FA and MD measures without reference to the vertex-weighting that was applied.

For comparison, these measures were also obtained from a transverse segment of the AC. The AC is a commissural fiber pathway—the function of which is not well understood—that provides interhemispheric connections between the temporal pole, the amygdala, the superior and inferior temporal gyri, and the parahippocampal gyrus (Demeter et al. 1990). Given that both the DHC and AC contain fibers that originate and cross in the parahippocampal gyrus, we restricted our AC analyses to those relatively “anterior projections” of this fiber bundle, which involve the temporal pole and amygdala. This was achieved by seeding tractography from an ROI manually drawn around the AC on a sagittal section 5 slices from the midline, where the AC is visible at the point it bifurcates the descending fornix columns (see Fig. 1*C*). This “SEED” ROI was initially drawn in the left hemisphere, and a corresponding AND ROI was placed at the same point in the right hemisphere. An exclusionary NOT ROI with whole-brain coverage was then drawn on an axial slice immediately above the AC. Another, covering the whole brain except the temporal lobes, was drawn on a coronal section immediately posterior to the rostrum of the corpus callosum. A final NOT ROI was drawn around the whole brain on a coronal section located just anterior to the pons. This procedure was repeated with the seed and the AND ROIs placed in the opposite hemispheres. The initial seed and AND ROIs were then used to extract a transverse segment of the AC from both reconstructions. Mean FA and MD metrics were extracted and combined using the above formula.

#### Statistical Analysis

Two-tailed Pearson correlation statistics were used to investigate the relationship between DT-MRI measures of DHC and AC microstructure (FA and MD) and performance in 3 standardized memory tasks (CPWM, PSMT, and LSWMT). Correlation statistics were computed with 1000 bootstrapped samples to derive 95% confidence intervals (CIs), and a Bonferroni–Holm step-down procedure was used to adjust derived *P* values for 6 structure–cognition correlations, separately for each tract (DHC and AC). Subjects in whom both the DHC and AC were successfully reconstructed were included in these analyses to enable fair comparisons between dependent correlations across these tracts. To test for differences between correlations across the DHC and AC, any significant structure–cognition associations identified in one tract were compared with the corresponding correlation in the other tract using one-tailed Steiger *Z* tests, which are reported alongside Cohen's *q* effect size measures (Cohen 1988). A significance threshold of *P* = 0.05 was used for all comparisons.

The same correlational approach was used to investigate the relationship between memory performance (in the CPWM, PSMT, and LSWMT) and the volume of individual temporal lobe regions including the amygdala, hippocampus, temporal pole, and entorhinal and parahippocampal cortices. Bilateral volume measurements were used to maximize statistical power, and *P* values were Bonferroni–Holm adjusted for 15 volume–cognition correlations. Regional gray matter volume measures are often confounded by interindividual differences in total ICV; we therefore used the following formula to adjust volume measurements for differences in ICV prior to any correlational analyses:

$$\begin{equation*} \textrm{Measure}_{\textrm{adjusted}} = \textrm{Measure}_{\textrm{raw}} - \beta (\textrm{ICV}_{\textrm{raw}} - \textrm{ICV}_{\textrm{mean}}) \end{equation*}$$

where ICV_raw_ refers to a subject's ICV estimate, ICV_mean_ refers to the mean ICV in the HCP dataset, and β refers to the slope of the regression line between ICV and the measure of interest (Voevodskaya et al. 2014).

**Figure 2:**
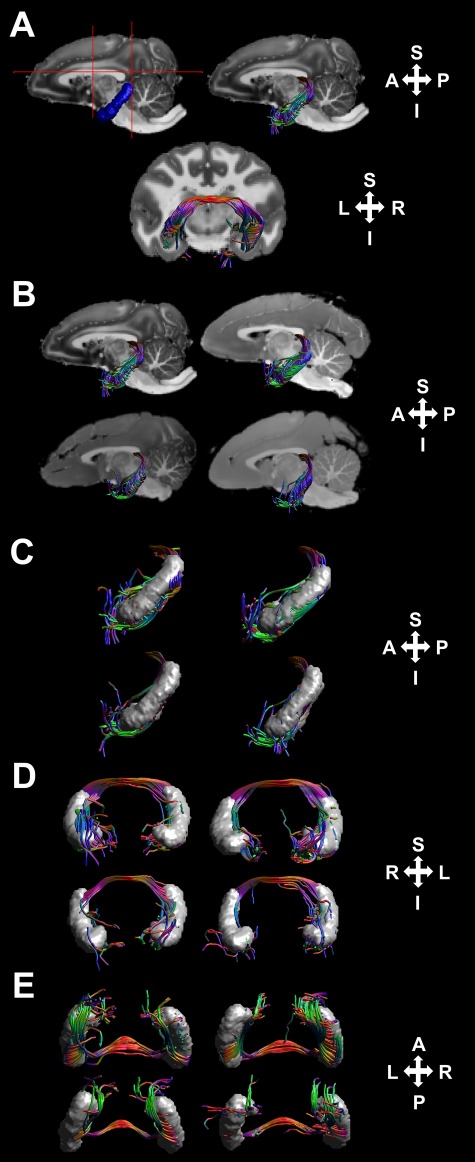
ROIs used to extract the DHC and the subsequent tract reconstructions. (*A*) The hippocampal (blue) and manually-drawn (red lines) ROIs used to extract the DHC in one representative specimen and the subsequent reconstructions shown over a midsagittal and coronal section from the corresponding *T*_1_-weighted image in a 0.5-mm^3^ native diffusion space. (*B*) The reconstructions in all 4 specimens. The DHC reconstructions illustrated from a left-lateral, anterior–posterior, and ventral perspective, alongside the anatomical hippocampal ROIs for spatial context (*C*, *D*, and *E*, respectively). Note that, for computational purposes, these renderings contain a one-eighth subsample of all reconstructed streamlines.

## Results

### Ex Vivo Nonhuman Primate MR Data

**Figure 3:**
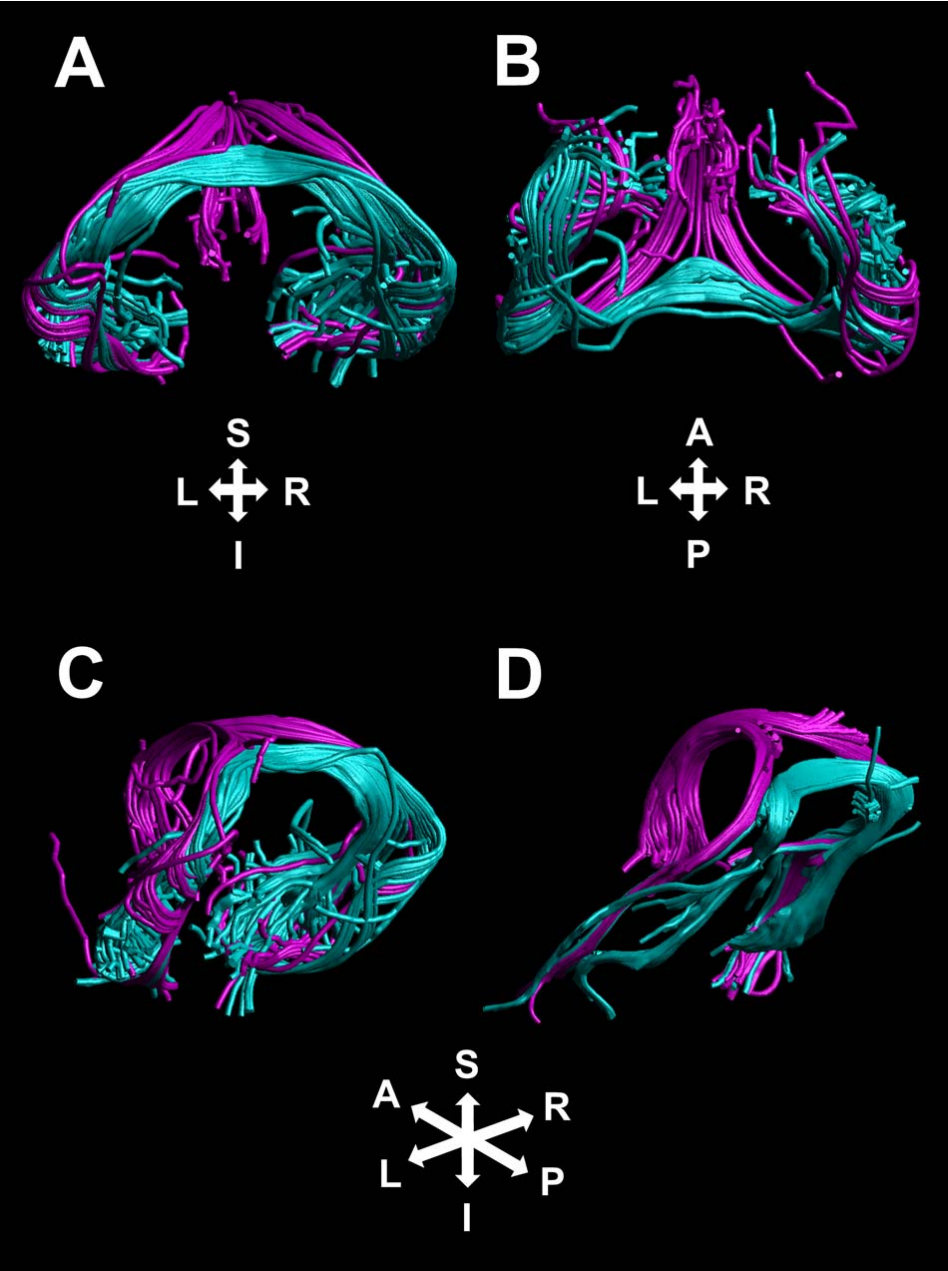
DHC (teal) and fornix (purple) streamlines reconstructed in representative cases. Streamlines corresponding to these tracts are shown for a representative ex vivo nonhuman primate specimen, shown from rear coronal oblique (*A*), ventral (*B*), and left-lateral oblique (*C*) perspectives. For comparison, streamlines corresponding to these 2 tracts are also shown for a representative HCP subject, from a left-lateral oblique perspective (*D*).

To demonstrate the feasibility of a white matter tractography approach for investigating the role of the DHC in human recognition memory, we first applied a multiple-ROI deterministic tractography protocol to diffusion- and *T*_1_-weighted images obtained from 4 ex vivo nonhuman primate brain specimens (see Methods and Figs 1 and 2*A*). In all 4 specimens, this revealed a large number of streamlines (mean = 2367.75, SD = 1383.791) that were broadly consistent with the known anatomy of the nonhuman primate DHC (see Fig. 2*B–E*). At the midline of the brain, the transverse portion of the DHC streamlines was situated along the anterior and inferior aspects of the rostral splenium of the corpus callosum; these streamlines did not extend anteriorly into the body of the fornix and cross at the point where the fornix body transitions into the anterior columns, where the VHC is known to cross the midline in nonhuman primates (Demeter et al. 1990). More laterally, these streamlines arched inferiorly towards the hippocampus and parahippocampal region. While a number of these streamlines progressed inferiorly towards regions along the parahippocampal gyrus (see Fig. 2*A*, right), consistent with the known anatomy, a number terminated in or around the hippocampus after having intersected our hippocampal ROIs. This finding highlights limitations in resolving crossing fiber populations with existing tractography techniques, and the fact that near the tail of the hippocampus, DHC fibers are known to merge with the alveus, which like the fimbria, then covers the hippocampus (Gloor et al. 1993). Indeed, Figure 3*A–C* shows the DHC reconstruction for a representative specimen alongside streamlines corresponding to the fornix, which were reconstructed for illustrative purposes using a multiple-ROI approach reported previously (Metzler-Baddeley et al. 2011); while the transverse portion of the DHC is readily differentiated, more laterally, many of the DHC streamlines become intermingled with those of the fornix as the latter covers the hippocampus. Nevertheless, these ex vivo DHC reconstructions suggest that white matter tractography can be used to detect and reconstruct interhemispheric DHC connections and that the transverse portion of these fiber pathway reconstructions, in particular, is well characterized and differentiated from the fornix. The reconstructions were similar in humans (Fig. 3*D*), so our subsequent quantitative analyses in human subjects were based on mean microstructure measures that were extracted from this transverse portion of the DHC (see Methods).

**Figure 4:**
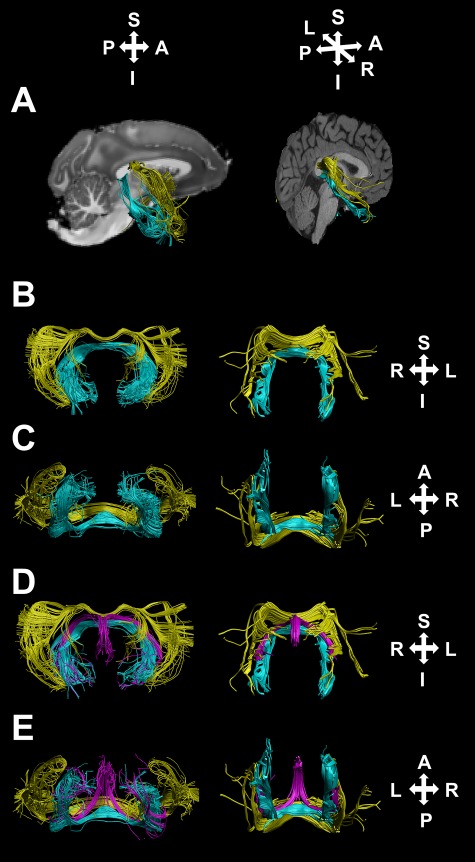
DHC (teal), tapetum (yellow), and fornix (purple) streamlines reconstructed in representative cases. (*A*) Streamlines corresponding to the DHC and tapetum in a nonhuman primate case (left) and an HCP subject (right), against a midsagittal *T*_1_-weighted image section. (*B*, *C*) The same streamlines from anterior-coronal and ventral perspectives, respectively. (*D*, *E*) The same streamlines alongside those corresponding to the fornix.

**Figure 5:**
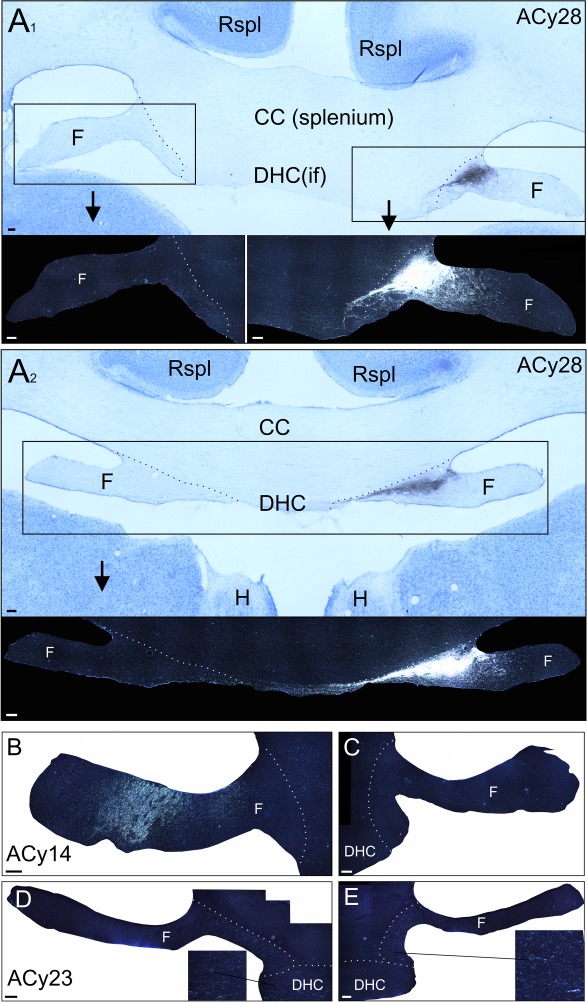
Bright- and dark-field photomicrographs of coronal sections taken at the level of the DHC, inferior to the corpus callosum. *A*_1_ contains a bright-field photomicrograph from case ACy28, which received a large tracer injection that filled much of the caudal hippocampus, including the subiculum. Dense anterograde label can be seen in the medial fornix alongside a lighter label seemingly entering the DHC at the level of the inferior forceps. The large majority of this label then turned rostral to occupy the medial half of the DHC, as seen in *A*_2_. *A*_2_ is from the same case but more anterior (at the level of the habenula). Some of the label in the DHC decussates to join the medial contralateral fornix. (*B*, *C*) Dark-field photomicrographs from case ACy14 (coronal section at the level of the splenium), which received a tracer injection centered in the rostral subiculum. A very clear label is present in the ipsilateral (*B*), but not contralateral (*C*), fornix, while no label is apparent in the DHC at the level of the inferior forceps. (*D*, *E*) Dark-field photomicrographs from case ACyF23, whose injection incorporated the caudal perirhinal and anterior parahippocampal cortices. Light label was evident in the most medial fornix, alongside labeled fibers in both the left and right DHC, within the inferior forceps. Magnified inserts are included in panels *A*_1_, *A*_2_, *D*, and *E*. All scale bars = 200 μm. Abbreviations: CC, corpus callosum; F, fornix; H, habenula; if, inferior forceps; Rspl, retrosplenial cortex.

Although the transverse portion of the DHC reconstructions was well differentiated from the fornix, at the midline of the living brain, fibers at the caudal border of the DHC are also known to be intermingled with those of the rostral splenium, which contains both the forceps major and the tapetum (Demeter et al. 1985, 1990; Gloor et al. 1993; Davis and Cabeza 2015). Our combined ROIs precluded an erroneous reconstruction of connections between bilateral occipital regions, which would correspond to the forceps major. The DHC does, however, have a similar morphology to the tapetum of the corpus callosum (Fig. 4), which, as it travels laterally and then ventrally from the splenium, forms part of the roof and lateral wall of the lateral ventricles and terminates in bilateral inferior temporal cortical regions (Demeter et al. 1990) and the paired caudate nuclei (Fernández-Miranda et al. 2008; Güngör et al. 2017). Unlike tapetum fibers, which form part of the roof and lateral wall of the lateral ventricles, the crus of the fornix forms the posterior medial margin of the lateral ventricles (Raslau et al. 2015), and the lateral portion of the DHC is, in turn, briefly attached to the inferior surface of the fornix crus (Gloor et al. 1993); the lateral portions of the DHC and tapetum are therefore spatially distinct and are well differentiated, as illustrated in Figure 4. The tapetum also crosses the midline of the brain at a level within the splenium that is superior to the DHC.

### Ex Vivo Nonhuman Primate Tract-Tracer Data

To help confirm the cortical origins of the interhemispheric DHC streamlines that were reconstructed in the previous analysis, we next examined sections (bright- and dark-field) taken from 3 cynomolgus monkeys that had previously received anterograde tracer injections in different locations within the MTL.

Case ACy28 received extensive injections of tracer that largely filled all fields of the posterior hippocampus, including the subiculum, and just reached the border with the adjacent presubiculum. The tracer did not extend into the parahippocampal cortices. As is evident from Figure 5*A*, very dense fiber labeling emerged from the hippocampus to fill much of the most medial fornix (Fig. 5*A*_1_). At the caudal part of the body of the fornix (at the level of the splenium and inferior forceps), a more ventral and medial subset of fibers seemed headed for the DHC (Fig. 5*A*_1_). In fact, the large majority of fibers turned rostrally to fill the most medial part of the anterior fornix. A small minority of fibers crossed in the more rostral DHC (Fig. 5*A*_2_), where they decussated within the fornix. Consequently, with the rostral fornix, the contralateral label was restricted to locations that mirrored the location of the signal in the ipsilateral fornix, although it was considerably lighter. Meanwhile, at the level of the splenium, just a few labeled fibers crossed in the DHC (inferior forceps; see Fig. 5*A*_1_).

In a case (ACy14) that received a much more restricted tracer injection in the rostral hippocampal formation, centered in the subiculum, dense labeling was present in the ipsilateral but not the contralateral fornix or the DHC (Fig. 5*B,C*). This pattern is consistent with previous research showing that the majority of fibers comprising the fornix originate in the subicular cortices and CA subregions of the hippocampal formation and that neither the fornix or the DHC supports interhemispheric connections between these regions (Saunders and Aggleton 2007).

By contrast, the case (ACyF23) that received an injection of tracer into the caudal perirhinal and rostral parahippocampal cortices contained label in both the left and right DHC but almost no label in either the ipsilateral or contralateral fornix (Fig. 5*D,E*). This distribution is consistent with DHC fibers originating in regions within the parahippocampal gyrus rather than the hippocampus proper. This set of findings highlight how the DHC is very largely populated by parahippocampal, rather than hippocampal, fibers.

**Figure 6:**
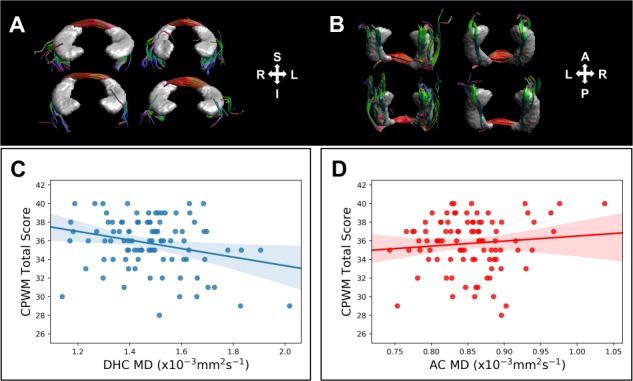
The DHC reconstructions in 4 representative HCP datasets and structure–cognition correlations reported in the text. DHC reconstructions are shown in the coronal (*A*) and axial (*B*) planes. The correlations between white matter MD and CPWM total scores in the DHC and AC are illustrated (in *C* and *D*, respectively). The best-fitting linear regression line is plotted alongside 95% CIs.

### In Vivo Human MR and Cognitive Data

#### Association between DHC microstructure and recognition memory performance

We derived diffusion tensor imaging metrics (FA and MD; Basser and Pierpaoli 2011) from the DHC in a subsample of 100 participants in the HCP for whom *T*_1_-weighted and DW-MRI data were available for analysis, along with performance in 3 standardized memory tasks: the PSMT (Dikmen et al. 2014), LSWMT (Tulsky et al. 2014), and CPWM task (Moore et al. 2015). This enabled us to investigate whether interindividual variation in DHC microstructure was correlated with memory performance. For comparison, these analyses were repeated in another commissure tract—the AC (see Methods).

Figure 6*A,B* illustrates the DHC reconstructions in 4 representative HCP subjects. Streamlines broadly consistent with the known anatomy of the DHC were successfully reconstructed in 96 subjects (96%). Similarly, streamlines consistent with AC anatomy were successfully reconstructed in 99 subjects (99%). Measures of DHC and AC microstructure (FA and MD) are reported in Table 1.

Cognitive performance in the CPWM, PSMT, and LSWMT is reported in Table 2. DT-MRI metrics for both the DHC and AC were available in 95 subjects, and a series of 2-tailed Pearson correlation analyses revealed a significant negative association between MD and CPWM performance in the DHC (*r* = −0.269, *P* = 0.048, 95% CI −0.499 to −0.017), which was not evident in the AC (*r* = 0.100, *P* = 1.0, 95% CI −0.123 to 0.297); further, these correlations were significantly different from one another (*Z* = −2.608, *P* = 0.009, *q* = 0.376; see Fig. 6*C,D*). The correlations between DHC MD and both PSMT and LSWMT performance were not statistically significant (*r* = −0.072, *P* = 1.0, 95% CI −0.260 to 0.096; *r* = −0.047, *P* = 0.649, 95% CI −0.260 to 0.159, respectively), although they were not significantly different from the association between DHC MD and CPWM performance (*Z* = −1.517, *P* = 0.065, *q* = 0.204; *Z* = −1.6, *P* = 0.055, *q* = 0.229, respectively). Across the DHC and AC, there were no other statistically significant structure–cognition associations (largest *r* = 0.211, *P* = 0.240, 95% CI 0.0–0.403). These findings imply a potential role for the DHC in CPWM performance—a standardized recognition memory test.

**Table 1:** Mean measures of DHC and AC microstructure in the HCP dataset (FA and MD) and N streamlines reconstructed

**Tract**	**Mean FA**	**Mean MD** (×10^−3^ mm^2^ s^−1^)	**Mean N streamlines**
DHC	0.318 (0.059)	1.478 (0.163)	1622.47 (1981.16)
AC	0.439 (0.05)	0.854 (0.051)	4102.68 (1896.59)

Note: Standard deviations are provided in parentheses.

**Table 2:** Mean performance in the CPWM, PSMT, and LSWMT

**CPWM**	**PSMT**	**LSWMT**
35.79 (2.78)	112.15 (14.34)	110.15 (11.49)

Note: Raw scores are reported for the CPWM, and scaled scores are reported for the PSMT and LSWMT. Standard deviations are provided in parentheses.

We explored the possibility that the association between DHC MD and CPWM performance was driven by the presence of a small number of particularly high or low DHC MD values. First, we ran an additional post hoc 2-tailed and nonparametric Spearman's rho correlation statistic, which is comparatively robust to outliers, and revealed a trend negative association between CPWM performance and DHC MD (*r_s_* = −0.197, *P* = 0.056, 95% CI −0.383 to 0.005). The removal of outliers should take into account biological interpretation and not be based solely on statistical tests (Schwarzkopf et al. 2012). We had no biological rationale for removing specific cases from our analyses (e.g., all reconstructions were consistent with known anatomy), and individuals with comparatively high or low DHC MD measurements are potentially—perhaps even especially—important and relevant biological observations. Nevertheless, inspection of a box plot in JASP (version 0.9) highlighted 2 individuals included in our initial comparisons as potential statistical outliers on the basis of relatively high DHC MD values; we removed these individuals from the dataset and performed an additional post hoc 2-tailed Pearson correlation statistic, which again revealed a trend negative association between DHC MD and CPWM performance(*r* = −0.202, *P* = 0.052, 95% CI −0.392 to 0.002). These post hoc tests highlight that, while including individuals with high DHC MD values increased the strength of the observed association between DHC MD and CPWM performance, their inclusion does not drive a statistical association for which there was not already a clear trend.

Finally, we ran a post hoc nondirectional Bayesian correlation test using JASP to quantify the evidence that the data provide for a relationship between DHC MD and CPWM performance. This analysis produces a Bayes factor, expressed as BF_10_, which grades the evidence that the data provide for this alternative (H1) compared with the null hypothesis (H0) on a continuous scale, along with a 95% Bayesian credibility interval (BCI). The resulting Bayes factor of BF_10_ = 3.907 (95% BCI = [−0.441, −0.069]) indicates that the data are 3.907 times as likely to have occurred under the alternative compared with the null. Furthermore, this BF_10_ > 3 indicates substantial evidence for the alternative over the null (Wetzels and Wagenmakers 2012).

#### Association between temporal regional volumes and memory

Using pre-existing regional volume estimates for our HCP subsample, we assessed whether the bilateral volumes of MTL gray matter regions, including the hippocampus, amygdala, temporal pole, and entorhinal and parahippocampal cortices, are also related to memory performance. These measures were first adjusted for differences in total ICV (see Methods) and are reported in Table 3. There was no significant association between performance in any of the cognitive tasks (CPWM, PSMT, or LSWMT) and the bilateral ICV-adjusted volumes of these temporal regions (largest *r* = −0.189, *P* = 0.885, 95% CI −0.368 to −0.004).

**Table 3:** Mean ICV and ICV-adjusted volumes of bilateral temporal regions

**Structure**	**Volume (mm** ^3^ **)**
Total ICV	1 587 548.46 (176 651.55)
Hippocampus	8856.07 (663.78)
Amygdala	3210.26 (310.89)
Entorhinal cortex	3460.21 (575.65)
Parahippocampal cortex	4391.78 (523.85)
Temporal pole	4682.52 (490.93)

Note: Standard deviations are provided in parentheses.

## Discussion

This study demonstrated that white matter tractography can be used to reconstruct the DHC in both nonhuman primates (ex vivo) and humans (in vivo) and that these reconstructions broadly conform to the known anatomy of this understudied commissural fiber bundle. That these connections are distinct from those comprising the adjoining fornix is supported by the differential pattern of labeling observed in 3 cynomolgus monkeys injected with anterograde tracer in either the subiculum of the hippocampal formation (dense labeling in the ipsilateral fornix); the caudal hippocampus, including almost all of the subiculum (dense label in the ipsilateral fornix extending into ipsilateral DHC with just a small minority crossing); or perirhinal/parahippocampal cortex (commissural label largely restricted to the DHC). Interindividual variation in the MD of the DHC reconstructions was also correlated with performance in a standardized recognition memory task—the CPWM. Importantly, this structure–cognition association was not evident in another commissural fiber bundle—the AC—implying a degree of specificity in the association between DHC microstructure and CPWM performance. The bilateral volumes of several temporal gray matter regions were not correlated with memory performance.

That our tractography approach affords reconstructions of the DHC across species is consistent with a preservation of DHC morphology across humans and nonhuman primates (Gloor et al. 1993). Wei et al. (2017) showed that a combination of manually and anatomically-defined ROIs, including the hippocampus, could be used to reconstruct the human DHC in vivo. Our results augment those findings by demonstrating that this approach is reliable for large datasets and by defining a specific combination of ROIs that yield DHC reconstructions that broadly reflect the known anatomy.

The DHC is frequently described as a component of the fornix that supports interhemispheric connections between the hippocampi (Raslau et al. 2015; Tubbs et al. 2015). While consistent with our DHC reconstructions, this anatomical interpretation is potentially misleading. Our anterograde tract-tracer data, for instance, highlight the distinct hippocampal and parahippocampal origins of the fibers comprising the fornix and DHC in nonhuman primates (Demeter et al. 1985, 1990). The present specimens were previously reported alongside others with more varied injections within the hippocampal formation and parahippocampal region; again, only in cases where injections involved parahippocampal regions was any incidental DHC labeling noted (Saunders and Aggleton 2007). The cortical origins of human DHC fibers have not been directly confirmed, but the human DHC may also connect parahippocampal regions rather than the hippocampi. Hippocampal ROIs can nevertheless be used to seed DHC tractography, as evidenced by our tract reconstructions, because across nonhuman primates and humans, DHC fibers originating in parahippocampal areas aggregate and travel towards the hippocampal tail through the alveus, which does itself cover the hippocampus (Gloor et al. 1993). The successful propagation of DHC streamlines from hippocampal ROIs may therefore reflect the contact between the alveus and hippocampus, and limitations in resolving crossing fiber populations associated with current tractography techniques (Jones et al. 2013), rather than any direct interhippocampal connectivity in the living brain. A proportion of those streamlines also terminated prematurely in the contralateral hippocampal ROIs, but several streamlines did nevertheless terminate in contralateral parahippocampal regions.

The hippocampus is also covered by the fimbria. Hippocampal contributions to the fornix enter the fornix crus via the alveus and then the fimbria; by contrast, DHC fibers travel through the alveus, but without traversing into the fimbria, they briefly take up a position on the inferior-caudal surface of the fornix crus before turning towards the midline (Gloor et al. 1993). Although the DHC and fornix crus are partially contiguous, their distinct cortical origins suggest that the DHC may play a unique—if complementary—role in mnemonic processing. The forebrain commissures enable an efficient coupling of events across hemispheres. As a result, congruent inputs to homologous regions across the 2 hemispheres are combined into a unitary percept, and items viewed in one visual field are quickly remembered when re-encountered in the other (Doty 2003). The DHC could therefore facilitate visual field integration and cross-hemisphere mnemonic processing. Bilateral parahippocampal contributions to specific memory processes may also be optimized through communication via the DHC.

Indeed, the association between DHC MD and CPWM performance highlights a potential role for the DHC in recognition memory. We were, however, limited to analyzing cognitive data from the HCP cognitive task battery, which is not necessarily optimized to investigate the role of the DHC in different memory processes. Further interpretation of the modest relationship between DHC MD and CPWM performance is therefore not straightforward. The CPWM is a recognition memory task in which participants must discriminate between novel and pre-exposed words but are not required to perform free recall of studied items. According to dual-process models of recognition memory (Aggleton and Brown 1999; Diana et al. 2007; Brown et al. 2010), performance in such tasks could be supported by a familiarity-based recognition memory process, which is dependent on parahippocampal regions within the MTL (particularly the perirhinal cortex), rather than the hippocampus, which is instead critical for successful performance in tasks that require conscious recollection (e.g., free recall). Our findings therefore tentatively suggest that the DHC may play a role in successful familiarity-based recognition memory. By contrast, the fornix supports hippocampal interactions with regions beyond the temporal lobe and has been more strongly implicated in recollection-based recognition memory (Rudebeck et al. 2009; Vann et al. 2009). The DHC and fornix may therefore play dissociable roles in these recognition memory processes.

CPWM performance is not, however, a process-pure measure of familiarity-based recognition memory. Although the CPWM places no explicit demands on recollection, this putatively distinct mnemonic process may also be recruited to aid performance. Furthermore, the association between DHC MD and CPWM performance was not significantly different to that between DHC MD and PSMT performance, and successful performance in the latter task may be more dependent upon recollection processes. To disentangle the specific memory processes that are partly dependent on DHC connections, future studies should employ a variety of recognition memory paradigms that place differential demands on familiarity- and recollection-based recognition memory, including both free-recall and forced-choice recognition tasks. Such studies will both augment the present findings and extend the conclusions that can be made about the role of the DHC in recognition memory.

While the CPWM employs verbal stimuli, the PSMT employs visual stimuli, albeit with additional verbal descriptors. The present association between DHC MD and CPWM performance was not significantly different to that between DHC MD and PSMT performance. Our study did not, therefore, reveal a differential role for the DHC in verbal compared with visual memory. Future research should employ matched verbal and visual memory paradigms to ascertain the extent to which interhemispheric mnemonic processing of such stimuli depends upon DHC connections. A considerable body of literature indicates a degree of hemispheric specialization in visual and verbal processing (Gross 1972; Papanicolaou et al. 2002; Nagel et al. 2013). While we identified an association between DHC microstructure and performance in one verbal recognition memory task, it is possible that DHC connections are particularly important for the mnemonic processing of task-relevant conjunctions of visual and verbal information.

There were no significant correlations between measures of either DHC or AC microstructure and performance in the LSWMT, implying that these tracts are not involved in working memory. However, the correlations between DHC MD and a) CPWM performance and b) LSWMT performance were not statistically different. Future research should include tasks outside the recognition memory domain to assess any role for the DHC in other forms of learning and memory. Our analyses also revealed no associations between memory measures and the bilateral volumes of temporal regions that are known to be connected via the DHC/AC (Klingler and Gloor 1960; Demeter et al. 1985, 1990). Based on findings from experiments in monkeys, a number of these regions were previously identified as components of a core MTL memory system supporting recognition memory performance (Zola-Morgan et al. 1989; Squire and Zola-Morgan 1991). Other investigators highlight distinct contributions from a smaller subset of MTL regions, including the hippocampus and perirhinal cortex (Aggleton and Brown 1999) and sometimes the parahippocampal cortex (Diana et al. 2007; Ranganath and Ritchey 2012; Diana 2016). Further investigations are required to understand the functional specialization within the MTL and the relationship between recognition memory performance, white matter microstructure, and gray matter macrostructure in this region.

Our results imply that the human DHC is not vestigial, which has implications for the treatment of several neurological conditions. The DHC could, for instance, be incorporated into models of the cognitive impact of resective MTL epilepsy surgeries (Trenerry et al. 1993; Dupont 2015). Intracranial electroencephalography studies indicate that a subset of seizures with a medial temporal onset have a pattern of contralateral spread to the hippocampus prior to involvement of the contralateral neocortex, potentially via the DHC (Gloor et al. 1993; Rosenzweig et al. 2011). Indeed, whether due to bilateral hippocampal pathology or seizure spread, voxel-based morphometry analyses have identified a cluster of voxels that incorporates the DHC, in which white matter volume is reduced in temporal lobe epilepsy cases with bilateral hippocampal sclerosis compared with healthy controls (Miró et al. 2015). Our tractography protocols offer a complimentary approach to investigating whether DHC microstructure is also compromised in epilepsy cases with mesial temporal sclerosis.

Tractography uses information about the directionality of water molecule diffusion to infer, discretely, the orientation of underlying tracts and reconstruct continuous streamlines in vivo. Tractography is, however, limited by the available MRI voxel resolution, which is inherently coarse compared with the dimensions of individual axons. Individual voxels can therefore contain multiple crossing fiber populations (Jeurissen et al. 2013; Jones et al. 2013). We used a tractography approach based on CSD to help resolve crossing fibers (Tournier et al. 2008). Nevertheless, owing to the “crossing fiber problem,” CSD-based tractography can still reconstruct both false-positive and false-negative connections (Maier-Hein et al. 2017). We therefore used complimentary tract-tracer data to interpret the anatomical connectivity implied by our DHC reconstructions. These techniques enable researchers to trace injections of radioactive amino acids from the origin of an axonal projection to its terminal, thereby providing a direct albeit ex vivo assessment of interregional connectivity (Oztas 2003). The use of different species for our nonhuman primate tractography and tract-tracer analyses (vervet and cynomolgus monkeys, respectively) is, however, a limitation of the current study. Nevertheless, both species are members of the *Cercopithecinae* subfamily of Old World monkeys, and their brain anatomy is considered to be very similar (Woods et al. 2011). Tract-tracer data obtained in one of these species can therefore aid in the interpretation of tractography results obtained in the other.

An advantage of our hypothesis-driven tractography approach is that, by constraining our quantitative analyses to 2 commissural tracts, we reduce the risk of reporting both false-positive effects in regions for which we have no specific predictions and false-negative effects when true structure–cognition relationships are obscured following corrections for large numbers of statistical comparisons. Another advantage of our tractography approach, in which we extract DT-MRI–based microstructural indices that are averaged over a tract-of-interest, is that it may be more sensitive to subtle microstructural differences that are distributed along the length of the tracts compared with voxel-based methods in which such differences must be clustered in order to detect a significant effect in group-level analyses (e.g., tract-based spatial statistics) (Smith et al. 2006). Voxel-based methods and metrics that take into account dispersed structural differences could, however, provide complimentary evidence of a role for the DHC in memory. Anatomical connectivity mapping (ACM) has been proposed as a method of quantifying the strength of connectivity of individual voxels with the rest of the brain (Bozzali et al. 2011). Within a tract, the ACM metric at a given voxel may be sensitive to structural differences further along that tract. Similar to our approach, an average or median ACM measure can also be derived for a given tract-of-interest and used in structure–cognition correlations (Lyksborg et al. 2014). ACM could potentially provide complimentary evidence of a role for the DHC in recognition memory.

We used deterministic rather than probabilistic CSD-based tractography, which can provide additional information about the reproducibility of pathways through the data between 2 ROIs. Further, probabilistic tractography results could arguably be thresholded to remove streamlines from voxels where the uncertainty is high. DHC fibers briefly traverse the alveus, however, and diffusion MRI measures at the available voxel resolution do not distinguish the alveus and fimbria–fornix (Amaral et al. 2018). The selection of a threshold to exclude erroneous fornix contributions to the lateral portion of the DHC reconstructions and the derived diffusivity metrics is therefore subjective. Rather than arbitrarily thresholding out such streamlines, our deterministic approach addresses potential concerns about the specificity of DHC diffusivity parameters by deriving these from only the medial portion of our DHC reconstructions, which is well differentiated from the fornix.

At the midline of the living brain, however, fibers at the caudal border of the DHC are also intermingled with those of the rostral splenium, for example, the inferior portion of the tapetum and the forceps major (Demeter et al. 1985, 1990). Given the limited available MRI voxel resolution and consequent risk of partial volume contamination, it is possible that the tapetum and forceps major may have contributed variance to the microstructure measures derived from the DHC segments. While we cannot definitively exclude this possibility, our ROIs precluded reconstruction of forceps major streamlines connecting bilateral occipital regions, and our DHC reconstructions were well differentiated from the tapetum.

Although DHC MD was correlated with CPWM performance, DHC FA was not. FA and MD are both affected by multiple axonal properties, including myelination, density, diameter, and configuration as well as partial volume interactions with tract size (Vos et al. 2011; Jones et al. 2013). It is therefore not possible to attribute differences between our FA/MD findings to a single white-matter subcomponent. Further, our quantitative analyses involved a comparison of these measures across the DHC and AC. These tracts have a comparable morphology, and both are positioned near regions with isotropic diffusivity characteristics, for example, ventricles. The AC is therefore a reasonable comparison commissure tract for the DHC. The AC is larger, however, consistent with the higher streamline counts in our AC reconstructions (Lamantia and Rakic 1990). Owing to the sensitivity of MD to partial volume interactions with tract size, the 2 tracts are also differentially susceptible to partial volume contamination with nearby cerebrospinal fluid, as reflected by the higher mean MD in the DHC compared with AC segments.

In summary, this is the first study to use cross-species anatomical evidence to highlight the DHC as a discrete tract in primates and to systematically reconstruct it using advanced tractography techniques. Reconstructions of the human and nonhuman primate DHC broadly conform to the known anatomy of this tract, affording investigations of the role of the DHC in learning and memory. Indeed, we are also the first to demonstrate a correlation between interindividual variation in the microstructure of in vivo DHC tract reconstructions and differences in a measure of recognition memory performance. Our understanding of the unique role of the DHC in human learning and memory, in both health and disease, is sparse, but our approach should help advance knowledge of those aspects of human memory that are partly dependent upon interhemispheric processing via the DHC.

## Funding

D.K.J. and G.D.P. are supported by a Wellcome Trust Investigator Award and a Wellcome Trust Strategic Award (096646/Z/11/Z; 104943/Z/14/Z; both awarded to D.K.J.). M.P. is currently supported by the Medical Research Council (MR/N01233X/1).

## Notes

Data used in the preparation of this work were obtained from the MGH-USC HCP database (https://ida.loni.usc.edu/login.jsp). The HCP project (principal investigators: Bruce Rosen, MD, PhD, Martinos Center at Massachusetts General Hospital; Arthur W. Toga, PhD, University of California, Los Angeles; Van J. Weeden, MD, Martinos Center at Massachusetts General Hospital) is supported by the National Institute of Dental and Craniofacial Research, the National Institute of Mental Health, and the National Institute of Neurological Disorders and Stroke. Collectively, the HCP is the result of efforts of co-investigators from the University of California, Los Angeles; Martinos Center for Biomedical Imaging at Massachusetts General Hospital; Washington University; and the University of Minnesota. *Conflict of Interest*: The authors declare no competing interests.

## Author Contributions

M.W. developed the initial concept for this study, and the subsequent study design was developed by M.P., D.K.J., and M.W. Ex vivo vervet monkey MR images were previously acquired by H.L., M.P., and T.B.D., who contributed these existing datasets for the present tractography analyses. J.A. obtained and contributed both bright- and dark-field images from existing ex vivo cynomolgus monkey specimens. M.P. and M.W. performed tractography analyses with input from G.D.P. and under the supervision of D.K.J. All statistical analyses were performed by M.P. and M.W. with input from D.K.J. All authors provided critical revision of the manuscript, thereby providing important intellectual content.

## References

[ref1] Aggleton JP 2008 EPS Mid-Career Award 2006. Understanding anterograde amnesia: disconnections and hidden lesions. Q J Exp Psychol (Hove). 61:1441–1471. 18671169 10.1080/17470210802215335

[ref2] Aggleton JP 2012 Multiple anatomical systems embedded within the primate medial temporal lobe: implications for hippocampal function. Neurosci Biobehav Rev. 36:1579–1596. 21964564 10.1016/j.neubiorev.2011.09.005

[ref3] Aggleton JP, Brown MW 1999 Episodic memory, amnesia, and the hippocampal-anterior thalamic axis. Behav Brain Sci. 22:425–444 discussion 444–489. 11301518

[ref4] Aggleton JP, Desimone R, Mishkin M 1986 The origin, course, and termination of the hippocampothalamic projections in the macaque. J Comp Neurol. 243:409–421. 3512627 10.1002/cne.902430310

[ref5] Amaral RSC, Park MTM, Devenyi GA, Lynn V, Pipitone J, Winterburn J, Chavez S, Schira M, Lobaugh NJ, Voineskos AN et al. 2018 Manual segmentation of the fornix, fimbria, and alveus on high-resolution 3T MRI: application via fully-automated mapping of the human memory circuit white and grey matter in healthy and pathological aging. Neuroimage. 170:132–150. 27765611 10.1016/j.neuroimage.2016.10.027

[ref6] Basser PJ, Pierpaoli C 2011 Microstructural and physiological features of tissues elucidated by quantitative-diffusion-tensor MRI. J Magn Reson. 213:560–570. 22152371 10.1016/j.jmr.2011.09.022

[ref7] Begré S, Kiefer C, Von Känel R, Frommer A, Federspiel A 2009 Rey Visual Design Learning Test performance correlates with white matter structure. Acta Neuropsychiatr. 21:67–74. 25384565 10.1111/j.1601-5215.2009.00361.x

[ref8] Bozzali M, Parker GJM, Serra L, Embleton K, Gili T, Perri R, Caltagirone C, Cercignani M 2011 Anatomical connectivity mapping: a new tool to assess brain disconnection in Alzheimer’s disease. Neuroimage. 54:2045–2051. 20828625 10.1016/j.neuroimage.2010.08.069

[ref9] Brown MW, Warburton EC, Aggleton JP 2010 Recognition memory: material, processes, and substrates. Hippocampus. 20:1228–1244. 20848602 10.1002/hipo.20858

[ref10] Clark CR, Geffen GM 1989 Corpus callosum surgery and recent memory: a review. Brain. 112:165–175. 2645017 10.1093/brain/112.1.165

[ref11] Cohen J 1988 Statistical power analysis for the behavioral sciences, Routledge Academic. Hillsdale (NJ): Lawrence Erlbaum Associates, Publishers.

[ref12] Cook PA, Bai Y, Nedjati-Gilani S, Seunarine KK, Hall MG, Parker GJ, Alexander DC 2006 Camino: open-source diffusion-MRI reconstruction and processing. Proceedings of the 14th Scientific Meeting of the International Society for Magnetic Resonance in Medicine; Seattle, WA 14 p.

[ref13] D’Esposito M, Verfaellie M, Alexander MP, Katz DI 1995 Amnesia following traumatic bilateral fornix transection. Neurology. 45:1546–1550. 7644056 10.1212/wnl.45.8.1546

[ref14] Davis SW, Cabeza R 2015 Cross-hemispheric collaboration and segregation associated with task difficulty as revealed by structural and functional connectivity. J Neurosci. 35:8191–8200. 26019335 10.1523/JNEUROSCI.0464-15.2015 PMC4444541

[ref15] Demeter S, Rosene DL, Van Hoesen GW 1985 Interhemispheric pathways of the hippocampal formation, presubiculum, and entorhinal and posterior parahippocampal cortices in the rhesus monkey: the structure and organization of the hippocampal commissures. J Comp Neurol. 233:30–47. 3980771 10.1002/cne.902330104

[ref16] Demeter S, Rosene DL, Van Hoesen GW 1990 Fields of origin and pathways of the interhemispheric commissures in the temporal lobe of macaques. J Comp Neurol. 302:29–53. 2086614 10.1002/cne.903020104

[ref17] Diana RA 2016 Parahippocampal cortex processes the nonspatial context of an event. Cereb Cortex. 27:1808–1816. 10.1093/cercor/bhw014 PMC6317502 26874181

[ref18] Diana RA, Yonelinas AP, Ranganath C 2007 Imaging recollection and familiarity in the medial temporal lobe: a three-component model. Trends Cogn Sci. 11:379–386. 17707683 10.1016/j.tics.2007.08.001

[ref19] Dikmen SS, Bauer PJ, Weintraub S, Mungas D, Slotkin J, Beaumont JL, Gershon R, Temkin NR, Heaton RK 2014 Measuring episodic memory across the lifespan: NIH toolbox picture sequence memory test. J Int Neuropsychol Soc. 20:611–619. 24960230 10.1017/S1355617714000460 PMC4254833

[ref20] Doty RW 2003 Unity from duality. Acta Neurobiol Exp (Wars). 63:163–170. 14518508 10.55782/ane-2003-1464

[ref21] Dupont S 2015 Imaging memory and predicting postoperative memory decline in temporal lobe epilepsy: insights from functional imaging. Rev Neurol (Paris). 4620:201–325. 10.1016/j.neurol.2014.12.001 25726354

[ref22] Dyrby TB, Baaré WFC, Alexander DC, Jelsing J, Garde E, Søgaard LV 2011 An ex vivo imaging pipeline for producing high-quality and high-resolution diffusion-weighted imaging datasets. Hum Brain Mapp. 32:544–563. 20945352 10.1002/hbm.21043 PMC6870191

[ref23] Dyrby TB, Innocenti GM, Bech M, Lundell H 2018 Validation strategies for the interpretation of microstructure imaging using diffusion MRI. Neuroimage. 182:62–79. 29920374 10.1016/j.neuroimage.2018.06.049

[ref24] Dyrby TB, Lundell H, Burke MW, Reislev NL, Paulson OB, Ptito M, Siebner HR 2014 Interpolation of diffusion weighted imaging datasets. Neuroimage. 103:202–213. 25219332 10.1016/j.neuroimage.2014.09.005

[ref25] Dyrby TB, Sogaard LV, Hall MG, Ptito M, Alexander DC 2013 Contrast and stability of the axon diameter index from microstructure imaging with diffusion MRI. Magn Reson Med. 70:711–721. 23023798 10.1002/mrm.24501 PMC4199276

[ref26] Fedorov A, Li X, Pohl KM, Bouix S, Styner M, Addicott M, Wyatt C, Daunais JB, Wells WM, Kikinis R 2011 Atlas-guided segmentation of vervet monkey brain MRI. Open Neuroimag J. 5:186–197. 22253661 10.2174/1874440001105010186 PMC3256578

[ref27] Fernández-Miranda JC, Rhoton AL, Álvarez-Linera J, Kakizawa Y, Choi C, De Oliveira EP 2008 Three-dimensional microsurgical and tractographic anatomy of the white matter of the human brain. Neurosurgery. 62:989–1028. 18695585 10.1227/01.neu.0000333767.05328.49

[ref28] Glasser MF, Sotiropoulos SN, Wilson JA, Coalson TS, Fischl B, Andersson JL, Xu J, Jbabdi S, Webster M, Polimeni JR et al. 2013 The minimal preprocessing pipelines for the Human Connectome Project. Neuroimage. 80:105–124. 23668970 10.1016/j.neuroimage.2013.04.127 PMC3720813

[ref29] Gloor P, Salanova V, Olivier A, Quesney LF 1993 The human dorsal hippocampal commissure. Brain. 116:1249–1273. 8221057 10.1093/brain/116.5.1249

[ref30] Gross MM 1972 Hemispheric specialization for processing of visually presented verbal and spatial stimuli. Percept Psychophys. 12:357–363.

[ref31] Güngör A, Baydin S, Middlebrooks EH, Tanriover N, Isler C, Rhoton AL 2017 The white matter tracts of the cerebrum in ventricular surgery and hydrocephalus. J Neurosurg. 126:945–971. 27257832 10.3171/2016.1.JNS152082

[ref32] Heilman KM, Sypert GW 1977 Korsakoff’s syndrome resulting from bilateral fornix lesions. Neurology. 27:490–493. 558556 10.1212/wnl.27.5.490

[ref33] Jenkinson M, Bannister P, Brady M, Smith S 2002 Improved optimization for the robust and accurate linear registration and motion correction of brain images. Neuroimage. 17:825–841. 12377157 10.1016/s1053-8119(02)91132-8

[ref34] Jenkinson M, Smith S 2001 A global optimisation method for robust affine registration of brain images. Med Image Anal. 5:143–156. 11516708 10.1016/s1361-8415(01)00036-6

[ref35] Jeurissen B, Leemans A, Jones DK, Tournier J-D, Sijbers J 2011 Probabilistic fiber tracking using the residual bootstrap with constrained spherical deconvolution. Hum Brain Mapp. 32:461–479. 21319270 10.1002/hbm.21032 PMC6869960

[ref36] Jeurissen B, Leemans A, Tournier J-D, Jones DK, Sijbers J 2013 Investigating the prevalence of complex fiber configurations in white matter tissue with diffusion magnetic resonance imaging. Hum Brain Mapp. 34:2747–2766. 22611035 10.1002/hbm.22099 PMC6870534

[ref37] Jeurissen B, Tournier J-D, Dhollander T, Connelly A, Sijbers J 2014 Multi-tissue constrained spherical deconvolution for improved analysis of multi-shell diffusion MRI data. Neuroimage. 103:411–426. 25109526 10.1016/j.neuroimage.2014.07.061

[ref38] Johnson H, Harris G, Williams K 2007 BRAINSFit: mutual information rigid registrations of whole-brain 3D images, using the Insight Toolkit. Insight J. 1–10. Available from: http://www.insight-journal.org/browse/publication/180 [Accessed: 04/07/2019].

[ref39] Jones DK, Knösche TR, Turner R 2013 White matter integrity, fiber count, and other fallacies: the do’s and don’ts of diffusion MRI. Neuroimage. 73:239–254. 22846632 10.1016/j.neuroimage.2012.06.081

[ref40] Klingler J, Gloor P 1960 The connections of the amygdala and of the anterior temporal cortex in the human brain. J Comp Neurol. 115:333–369. 13756891 10.1002/cne.901150305

[ref41] Lamantia A-S, Rakic P 1990 Cytological and quantitative characteristics of four cerebral commissures in the rhesus monkey. J Comp Neurol. 291:520–537. 2329189 10.1002/cne.902910404

[ref42] Leemans A, Jeurissen B, Sijbers J, Jones D 2009 ExploreDTI: a graphical toolbox for processing, analyzing, and visualizing diffusion MR data. Proceedings of the 17th Scientific Meeting International Society for Magnetic Resonance in Medicine. 17:3537.

[ref43] Lyksborg M, Siebner HR, Sørensen PS, Blinkenberg M, Parker GJM, Dogonowski AM, Garde E, Larsen R, Dyrby TB 2014 Secondary progressive and relapsing remitting multiple sclerosis leads to motor-related decreased anatomical connectivity. PLoS One. 9(4): e95540. 10.1371/journal.pone.0095540 PMC3991654 24748023

[ref44] Mahut H, Moss M, Zola-Morgan S 1981 Retention deficits after combined amygdalo-hippocampal and selective hippocampal resections in the monkey. Neuropsychologia. 19:201–225. 7254500 10.1016/0028-3932(81)90105-6

[ref45] Maier-Hein KH, Neher PF, Houde J-C, Côté M-A, Garyfallidis E, Zhong J, Chamberland M, Yeh F-C, Lin Y-C, Ji Q et al. 2017 The challenge of mapping the human connectome based on diffusion tractography. Nat Commun. 8:1349. 29116093 10.1038/s41467-017-01285-x PMC5677006

[ref46] Mark LP, Daniels DL, Naidich TP, Yetkin Z, J a B 1993 Anatomic moment—the hippocampus. Am J Neuroradiol. 14:709–712. 8517363 PMC8333373

[ref47] Metzler-Baddeley C, Jones DK, Belaroussi B, Aggleton JP, O’Sullivan MJ 2011 Frontotemporal connections in episodic memory and aging: a diffusion MRI tractography study. J Neurosci. 31:13236–13245. 21917806 10.1523/JNEUROSCI.2317-11.2011 PMC6623273

[ref48] Miró J, Gurtubay-Antolin A, Ripollés P, Sierpowska J, Juncadella M, Fuentemilla L, Sánchez V, Falip M, Rodríguez-Fornells A 2015 Interhemispheric microstructural connectivity in bitemporal lobe epilepsy with hippocampal sclerosis. Cortex. 67:106–121. 25955498 10.1016/j.cortex.2015.03.018

[ref49] Moore TM, Reise SP, Gur RE, Hakonarson H, Gur RC 2015 Psychometric properties of the Penn Computerized Neurocognitive Battery. Neuropsychology. 29:235–246. 25180981 10.1037/neu0000093 PMC4345134

[ref50] Moss M, Mahut H, Zola-Morgan S 1981 Concurrent discrimination learning of monkeys after hippocampal, entorhinal, or fornix lesions. J Neurosci. 1:227–240. 7264718 10.1523/JNEUROSCI.01-03-00227.1981 PMC6564115

[ref51] Mugler JP, Brookeman JR 1990 Three-dimensional magnetization-prepared rapid gradient-echo imaging (3D MP RAGE). Magn Reson Med. 15:152–157. 2374495 10.1002/mrm.1910150117

[ref52] Nagel BJ, Herting MM, Maxwell EC, Bruno R, Fair D 2013 Hemispheric lateralization of verbal and spatial working memory during adolescence. Brain Cogn. 82:58–68. 23511846 10.1016/j.bandc.2013.02.007 PMC3652620

[ref53] Oztas E 2003 Neuronal tracing. Neuroanatomy. 2:2–5.

[ref54] Papanicolaou AC, Simos PG, Castillo EM, Breier JI, Katz JS, Wright AA 2002 The hippocampus and memory of verbal and pictorial material. Learn Mem. 9:99–104. 12074997 10.1101/lm.44302 PMC182590

[ref55] Patenaude B, Smith SM, Kennedy DN, Jenkinson M 2011 A Bayesian model of shape and appearance for subcortical brain segmentation. Neuroimage. 56:907–922. 21352927 10.1016/j.neuroimage.2011.02.046 PMC3417233

[ref56] Paxinos G, Xu-Feng H, Petrides M, Toga AW 2009 The rhesus monkey brain in stereotaxic coordinates. 2nd ed. Amsterdam: Academic Press.

[ref57] Phelps EA, Hirst W, Gazzaniga MS 1991 Deficits in recall following partial and complete commissurotomy. Cereb Cortex. 1:492–491. 1822754 10.1093/cercor/1.6.492

[ref58] Pohl KM, Bouix S, Nakamura M, Rohlfing T, McCarley RW, Kikinis R, Grimson WEL, Shenton ME, Wells WM 2007 A hierarchical algorithm for MR brain image parcellation. IEEE Trans Med Imaging. 26:1201–1212. 17896593 10.1109/TMI.2007.901433 PMC2768067

[ref59] Powell HWR, Guye M, Parker GJM, Symms MR, Boulby P, Koepp MJ, Barker GJ, Duncan JS 2004 Noninvasive in vivo demonstration of the connections of the human parahippocampal gyrus. Neuroimage. 22:740–747. 15193602 10.1016/j.neuroimage.2004.01.011

[ref60] Ranganath C, Ritchey M 2012 Two cortical systems for memory-guided behaviour. Nat Rev Neurosci. 13:713. 22992647 10.1038/nrn3338

[ref61] Raslau FD, Augustinack JC, Klein AP, Ulmer JL, Mathews VP, Mark LP 2015 Memory part 3: the role of the fornix and clinical cases. Am J Neuroradiol. 36:1604–1608. 26045575 10.3174/ajnr.A4371 PMC7968751

[ref62] Rosenzweig I, Beniczky S, Brunnhuber F, Alarcon G, Valentin A 2011 The dorsal hippocampal commissure: when functionality matters. J Neuropsychiatry Clin Neurosci. 23:E45–E48. 21948928 10.1176/jnp.23.3.jnpe45

[ref63] Rudebeck SR, Scholz J, Millington R, Rohenkohl G, Johansen-Berg H, Lee ACH 2009 Fornix microstructure correlates with recollection but not familiarity memory. J Neurosci. 29:14987–14992. 19940194 10.1523/JNEUROSCI.4707-09.2009 PMC2825810

[ref64] Saunders RC, Aggleton JP 2007 Origin and topography of fibers contributing to the fornix in macaque monkeys. Hippocampus. 17:396–411. 17372974 10.1002/hipo.20276

[ref65] Schwarzkopf D, Haas B de, Rees G 2012 Better ways to improve standards in brain–behavior correlation analysis. Front Hum Neurosci. 6:200. 22811662 10.3389/fnhum.2012.00200 PMC3397314

[ref66] Smith SM, Jenkinson M, Johansen-Berg H, Rueckert D, Nichols TE, Mackay CE, Watkins KE, Ciccarelli O, Cader MZ, Matthews PM et al. 2006 Tract-based spatial statistics: voxelwise analysis of multi-subject diffusion data. Neuroimage. 31:1487–1505. 16624579 10.1016/j.neuroimage.2006.02.024

[ref67] Smith SM, Jenkinson M, Woolrich MW, Beckmann CF, Behrens TEJ, Johansen-Berg H, Bannister PR, De Luca M, Drobnjak I, Flitney DE et al. 2004 Advances in functional and structural MR image analysis and implementation as FSL. Neuroimage. 23:208–219. 10.1016/j.neuroimage.2004.07.051 15501092

[ref68] Sotiropoulos SN, Moeller S, Jbabdi S, Xu J, Andersson JL, Auerbach EJ, Yacoub E, Feinberg D, Setsompop K, Wald LL et al. 2013 Effects of image reconstruction on fiber orientation mapping from multichannel diffusion MRI: reducing the noise floor using SENSE. Magn Reson Med. 70:1682–1689. 23401137 10.1002/mrm.24623 PMC3657588

[ref69] Squire LR, Zola-Morgan S 1991 The medial temporal lobe memory system. Science. 253:1380–1386. 1896849 10.1126/science.1896849

[ref70] Tournier J-D, Yeh C-H, Calamante F, Cho K-H, Connelly A, Lin C-P 2008 Resolving crossing fibres using constrained spherical deconvolution: validation using diffusion-weighted imaging phantom data. Neuroimage. 42:617–625. 18583153 10.1016/j.neuroimage.2008.05.002

[ref71] Trenerry MR, Jack CR, Ivnik RJ, Sharbrough FW, Cascino GD, Hirschorn KA, Marsh WR, Kelly PJ, Meyer FB 1993 MRI hippocampal volumes and memory function before and after temporal lobectomy. Neurology. 43:1800–1805. 8414035 10.1212/wnl.43.9.1800

[ref72] Tubbs RS, Bosmia AN, Gupta T, Chawla K, Loukas M, Sahni D, Cohen-Gadol AA 2015 The enigmatic psalterium: a review and anatomic study with relevance to callosotomy procedures. Oper Neurosurg. 11:322–328. 10.1227/NEU.0000000000000747 25830602

[ref73] Tulsky DS, Carlozzi N, Chiaravalloti ND, Beaumont JL, Kisala PA, Mungas D, Conway K, Gershon R 2014 NIH toolbox cognition battery (NIHTB-CB): list sorting test to measure working memory. J Int Neuropsychol Soc. 20:599–610. 24959983 10.1017/S135561771400040X PMC4426848

[ref74] Turner BH, Mishkin M, Knapp ME 1979 Distribution of the anterior commissure to the amygdaloid complex in the monkey. Brain Res. 162:331–337. 104777 10.1016/0006-8993(79)90293-2

[ref75] Van Essen DC, Smith SM, Barch DM, Behrens TEJ, Yacoub E, Ugurbil K 2013 The WU-Minn Human Connectome Project: an overview. Neuroimage. 80:62–79. 23684880 10.1016/j.neuroimage.2013.05.041 PMC3724347

[ref76] Vann SD, Tsivilis D, Denby CE, Quamme JR, Yonelinas AP, Aggleton JP, Montaldi D, Mayes AR 2009 Impaired recollection but spared familiarity in patients with extended hippocampal system damage revealed by 3 convergent methods. Proc Natl Acad Sci. 106:5442–5447. 19289844 10.1073/pnas.0812097106 PMC2664061

[ref77] Voevodskaya O, Simmons A, Nordenskjöld R, Kullberg J, Ahlström H, Lind L, Wahlund L-O, Larsson E-M, Westman E 2014 The effects of intracranial volume adjustment approaches on multiple regional MRI volumes in healthy aging and Alzheimer’s disease. Front Aging Neurosci. 6:264. 25339897 10.3389/fnagi.2014.00264 PMC4188138

[ref78] Vos SB, Jones DK, Viergever MA, Leemans A 2011 Partial volume effect as a hidden covariate in DTI analyses. Neuroimage. 55:1566–1576. 21262366 10.1016/j.neuroimage.2011.01.048

[ref79] Wei PH, Mao ZQ, Cong F, Yeh FC, Wang B, Ling ZP, Liang SL, Chen L, Yu XG 2017 In vivo visualization of connections among revised Papez circuit hubs using full q-space diffusion spectrum imaging tractography. Neuroscience. 357:400–410. 28411159 10.1016/j.neuroscience.2017.04.003

[ref80] Weintraub S, Dikmen SS, Heaton RK, Tulsky DS, Zelazo PD, Bauer PJ, Carlozzi NE, Slotkin J, Blitz D, Wallner-Allen K et al. 2013 Cognition assessment using the NIH Toolbox. Neurology. 80:S54–S64. 23479546 10.1212/WNL.0b013e3182872ded PMC3662346

[ref81] Wetzels R, Wagenmakers E-J 2012 A default Bayesian hypothesis test for correlations and partial correlations. Psychon Bull Rev. 19:1057–1064. 22798023 10.3758/s13423-012-0295-x PMC3505519

[ref82] Woods RP, Fears SC, Jorgensen MJ, Fairbanks LA, Toga AW, Freimer NB 2011 A web-based brain atlas of the vervet monkey, Chlorocebus aethiops. Neuroimage. 54:1872–1880. 20923706 10.1016/j.neuroimage.2010.09.070 PMC3008312

[ref83] Yogarajah M, Powell HWR, Parker GJM, Alexander DC, Thompson PJ, Symms MR, Boulby P, Wheeler-Kingshott CA, Barker GJ, Koepp MJ et al. 2008 Tractography of the parahippocampal gyrus and material specific memory impairment in unilateral temporal lobe epilepsy. Neuroimage. 40:1755–1764. 18314352 10.1016/j.neuroimage.2007.12.046 PMC2330063

[ref84] Zola-Morgan S, Squire LR, Amaral DG, Suzuki WA 1989 Lesions of perirhinal and parahippocampal cortex that spare the amygdala and hippocampal formation produce severe memory impairment. J Neurosci. 9:4355–4370. 2593004 10.1523/JNEUROSCI.09-12-04355.1989 PMC6569635

